# Biochemical pathways mediated by KLK6 protease in breast cancer

**DOI:** 10.1002/1878-0261.12493

**Published:** 2019-09-30

**Authors:** Georgios Pampalakis, Eleni Zingkou, Konstantinos Gus Sidiropoulos, Eleftherios P. Diamandis, Vassilis Zoumpourlis, George M. Yousef, Georgia Sotiropoulou

**Affiliations:** ^1^ Department of Pharmacy School of Health Sciences University of Patras Rion‐Patras Greece; ^2^ The Keenan Research Center in the Li Ka Shing Knowledge Institute Department of Laboratory Medicine St. Michael's Hospital Toronto Canada; ^3^ Department of Laboratory Medicine and Pathobiology University of Toronto Canada; ^4^ National Hellenic Research Foundation Athens Greece

**Keywords:** apoptosis, KLK6, proteomics, S100A, triple‐negative breast cancer

## Abstract

Kallikrein‐related peptidase 6 (KLK6) is a serine protease normally expressed in mammary tissue and aberrantly regulated in breast cancer. At physiological levels, KLK6 functions as a suppressor of breast cancer, while its aberrant overexpression (> 50‐fold higher than normal) is characteristic of a subset of breast cancers and has been linked to accelerated growth of primary breast tumors in severe combined immunodeficiency mice (Pampalakis *et al. Cancer Res* 2009, 69, 3779). Here, we investigated the molecular mechanisms underlying the concentration‐dependent functions of KLK6 by comparing MDA‐MB‐231 stable transfectants expressing increasing levels of KLK6 in *in vitro* and *in vivo* tumorigenicity assays (soft agar, xenograft growth, tail vein metastasis). Quantitative proteomics was applied to identify proteins that are altered upon re‐expression of KLK6 in MDA‐MB‐231 at normal or constitutive levels. Overexpression of KLK6 is associated with increased metastatic ability of breast cancer cells into lungs, increased expression of certain S100 proteins (S100A4, S100A11) and keratins (KRT), and downregulation of the apoptosis‐related proteases CASP7 and CASP8, and RABs. On the other hand, KLK6 re‐expression at physiological levels leads to inhibition of lung metastases associated with suppression of S100 proteins (S100A4, S100A10, S100A13, S100A16) and induced CASP7 and CASP8 expression. As this is the first report that KLK6 expression is associated with S100 proteins, caspases, RABs, and KRTs, we validated this finding in clinical datasets. By integrating proteomics and microarray data from breast cancer patients, we generated two composite scores, KLK6 + S100B‐S100A7 and KLK6 + S100B‐S100A14‐S100A16, to predict long‐term survival of breast cancer patients. We present previously unknown pathways implicating KLK6 in breast cancer. The findings promise to aid our understanding of the functional roles of KLK6 in breast cancer and may yield new biomarkers for the cancer types in which KLK6 is known to be aberrantly upregulated.

**Abbreviations**
EMT: epithelial‐to‐mesenchymal transition
IPA: Ingenuity Pathway Analysis
KLK: kallikrein‐related peptidase
KRT: keratin
PBST: PBS with 0.05% Tween‐20
SCID: severe combined immunodeficiency
SFCM: serum‐free conditioned medium
TCGA: The Cancer Genome Atlas
TNBC: triple‐negative breast cancer

## 1 Introduction

Breast cancer is the most frequent cancer in women and second to all cancers worldwide. With an estimated 2.09 million cases diagnosed per year (data of year 2018), it ranks forth as cause of death from cancer (Bray *et al*., 2018). Based on microarray profiling data, breast tumors were classified into the following five distinct subtypes: triple‐negative basal‐like (TNBC), luminal A, luminal B, ErbB2‐overexpressing, and normal‐like (Perou *et al*., 2000; Sørlie *et al*., 2001). TNBCs are of poor prognosis; consequently, identification of new markers for early detection and/or monitoring is of great importance. Basal‐like breast cancers are further subdivided into basal A and B subtypes based on gene expression (Kao *et al*., 2009; Neve *et al*., 2006). The basal B subtype is recapitulated by several of the highly invasive cell lines, such as the MDA‐MB‐231, with manifested epithelial‐to‐mesenchymal transition (EMT) features (Kao *et al*., 2009). Since proteins are directly linked to functions and phenotypes, proteomic analysis of breast cancers could provide novel biomarkers and/or therapeutic targets (Kulasingam and Diamandis, 2007; Lawrence *et al*., 2015).

Kallikrein‐related peptidases (KLKs) constitute a family of 15 serine proteases with trypsin‐ or chymotrypsin‐like activity representing the largest cluster of serine proteases in the human genome. Certain KLKs are aberrantly expressed in various cancers, including breast cancer, and may have utility as biomarkers for diagnosis, prognosis, and response to therapy (Sotiropoulou *et al*., 2009; Stefanini *et al*., 2015). KLKs play instrumental roles in skin disorders, inflammation, and neurodegeneration; thus, they represent valuable druggable targets (Sotiropoulou and Pampalakis, 2012). Importantly, KLKs are emerging regulators of immune function (Sotiropoulou and Pampalakis, 2010). In particular, KLK6 was originally discovered as being highly upregulated in a primary breast tumor but inactivated in the corresponding lung metastases (Anisowicz *et al*., 1996). KLK6 is synthesized as an inactive zymogen that can autoactivate, and the active enzyme is capable to self‐inactivate by internal cleavage at arginine 80 (Bayés *et al*., 2004; Magklara *et al*., 2003), and its expression is regulated by multiple promoters and different alternatively spliced variants have been identified (Adamopoulos *et al*., 2017; Pampalakis *et al*., 2004). In human breast cancer cells, KLK6 expression is silenced via promoter methylation (Pampalakis and Sotiropoulou, 2006; Pampalakis *et al*., 2009). When KLK6 was re‐expressed in KLK6‐negative MDA‐MB‐231 cells at physiological levels (i.e., those produced in normal mammary tissue), it inhibited tumorigenicity *in vitro* and *in vivo*. Intriguingly, aberrant overexpression (at levels > 50‐fold higher than normal) of KLK6 resulted in accelerated growth of primary tumors in severe combined immunodeficiency (SCID) mice, indicating that overexpression of KLK6 promotes tumorigenesis. To delineate the molecular mechanisms underlying these concentration‐dependent effects of KLK6 on the malignant phenotype, we engineered MDA‐MB‐231 transfectant clones that stably express increasing levels of KLK6, including a clone that expresses intermediate levels (between normal and overexpression), and studied primary tumor growth following injection of these clones in SCID mice and their lung metastatic potential. We show that KLK6 inhibits both primary tumor growth and lung metastases only when re‐expressed at normal levels. The proteome of parental (PAR) and engineered MDA‐MB‐231 cells expressing low or high KLK6 levels was analyzed by HPLC‐MS/MS to identify putative molecular pathways underlying the observed, tumor suppression or promotion, respectively. The findings were validated in clinical samples, and two new combined scores for survival prediction are proposed.

## 2 Materials and methods

### 2.1 Materials

All chemicals were obtained from Sigma (St. Louis, MO, USA) or Merck (Darmstadt, Germany). Antibodies were as follows: anti‐α‐tubulin (T5168) and anti‐destrin (D8815) from Sigma; anti‐p65 (8242P), anti‐MMP2 (D8N9Y, 13132), and anti‐MMP9 (G657, 2270) from Cell Signaling (Danvers, MA, USA); and anti‐p53, kindly provided by Bořivoj Vojtěšek (Masaryk Memorial Cancer Institute, Czech Republic). Anti‐mouse and anti‐rabbit antibodies were obtained from Sigma and Millipore (Burlington, MA, USA), respectively.

### 2.2 Cell culture and transfection

MDA‐MB‐231 cells were maintained in RPMI supplemented with 10% FBS and antibiotics (penicillin/streptomycin). The pcDNA3.1(+)/preproKLK6 plasmid was transfected into MDA‐MB‐231 cells with PolyFect (Qiagen, Valencia, CA, USA). Cells were selected with G418 (0.5 mg·mL^−1^) in RPMI supplemented with 10% FBS. After 3 weeks, colonies were picked and grown individually in 96‐well plates in complete medium supplemented with G418 (0.5 mg·mL^−1^).

### 2.3 Soft agar assay

The assay was conducted as described previously (Pampalakis *et al*., 2009). Briefly, 10^5^ cells per well (six‐well plate) were plated in 0.3% agar (top layer) on a bottom layer of 0.5% agar. After 2 weeks, colonies were stained with 3‐(4,5‐dimethyl‐2‐thiazolyl)‐2,5‐diphenyl‐2H‐tetrazolium bromide and photographed.

### 2.4 *In vivo* tumorigenicity

Cells were trypsinized and resuspended in PBS. A total of 2 × 10^6^ cells resuspended in 100 μL PBS were orthotopically implanted into the mammary fat pad of 6‐ to 8‐week‐old SCID mice. Mice were observed on alternate days for the appearance of tumors. All experiments involving mice were performed in accordance with EU and national legislation and with the guidelines of the National Hellenic Research Foundation for ethics of animal experimentation.

### 2.5 Tail vein model

Cells were trypsinized and resuspended in PBS. A total of 2 × 10^6^ cells in 100 μL PBS were injected via the tail vein. After 8 weeks, mice were sacrificed and their lungs were removed and examined for the presence of visible metastases.

### 2.6 Cell lysates

Cells were cultured in T175 flasks to 70% confluence, then the medium was removed, and cells were washed with PBS and serum‐free conditioned medium (SFCM) three times, respectively, and incubated with SFCM for 48 h. Cells were scraped on ice‐cold PBS, harvested by centrifugation, and lysed in 50 mm Tris/HCl (pH 7.5), 100 mm NaCl, and 10 mm EDTA containing 1% NP‐40 for 30 min on ice. The lysates were recovered by centrifugation at 14 000 ***g*** for 10 min and used immediately. Protein concentration was measured with the Bradford assay (Bio‐Rad, Hercules, CA, USA) using bovine serum albumin as standard.

### 2.7 SDS/PAGE

Nine hundred micrograms of total protein extracts was treated with loading buffer under reducing conditions (β‐mercaptoethanol) and resolved on a 35‐cm‐long 12% SDS/PAGE. Bands were visualized with Coomassie Brilliant Blue R250 staining. Stained lanes were excised in 23 pieces and stored in 0.1% CH_3_COOH until used for *in‐gel* trypsin digestion and mass spectrometry.

### 2.8 Trypsin digestion

Gels were washed and destained in acetonitrile. Proteins were reduced and alkylated as described (Planque *et al*., 2009). The gels were incubated with trypsin in 10 mm ammonium bicarbonate. Peptide fragments were extracted and dried. The peptides were resuspended in buffer A (95% water, 0.1% formic acid, 5% acetonitrile, 0.02% trifluoroacetic acid) before loaded onto the HPLC column.

### 2.9 Mass spectrometry

The HPLC system used a 5‐cm C18 column (inner diameter of 75 μm) with an 8‐μm tip and was coupled to an LTQ Orbitrap XL ThermoFisher (Waltham, MA, USA) mass spectrometer, using a nanoelectrospray ionization source in a data‐dependent mode. Samples were run on a gradient (buffer A: 95% water, 0.1% formic acid, 5% acetonitrile, 0.02% trifluoroacetic acid; buffer B: 90% acetonitrile, 0.1% formic acid, 10% water, 0.02% trifluoroacetic acid). The eluted peptides were subjected to one full MS1 scan in the Orbitrap at 60 000 resolution, followed by six MS2 scans in the linear ion trap. The charges states of + 1 and > 4 as well as unassigned charges states were not subjected to MS/MS fragmentation.

### 2.10 Protein identification

The RAW files were uploaded on mascot daemon 2.2, and extractmsn (Matrix Science Inc, Boston, MA, USA) was used to generate DAT and then Mascot files. The Mascot files were searched on Global Proteome Machine Manager (2006.06.01) against the concatenated nonredundant IPI. The parameters were as follows: minimum mass, 300 Da; maximum mass, 4000 Da; automatic precursor charge selection; minimum peaks, 10 per MS/MS scan for acquisition; and minimum scans per group, 1. The false discovery rate for peptides was 5.6% and for proteins 2.9%.

### 2.11 Bioinformatics

Proteomic analysis data were processed with scaffold (Proteome Software Inc, Portland, OR, USA). Average linkage hierarchical clustering of protein profile was performed with cluster, and the results were displayed with treeview (http://rana.lbl.gov/EisenSoftware.htm) (Eisen *et al*., 1998). For pathway analysis, ingenuity pathway analysis (ipa) software (Ingenuity Systems, Mountain View, CA, USA) was used. The set of proteins differentially expressed in C5 and C28 was imported to ipa for analysis using 70 proteins as default.

### 2.12 Western blot analysis

Cell lysates were separated by SDS/PAGE, transferred onto poly(vinylidene difluoride) membranes, blocked with 5% nonfat dried milk in PBS, and incubated with primary antibodies (1 : 1000) in 1% nonfat dried milk in PBS with 0.05% Tween‐20 (PBST) for 1 h at room temperature. Then, the membranes were washed with PBS and incubated with secondary antibody (1 : 2500) in 1% nonfat dried milk in PBST. Finally, the membranes were washed four times in PBST and antibody‐specific signals were detected with West Pico enhanced chemiluminescence Pierce (ThermoFisher).

### 2.13 Gel zymography

Serum‐free conditioned media were concentrated to 1/5 of the original volume with Amicon (Millipore) spin filters with a 10‐kDa cutoff membrane and separated on SDS/PAGE containing 0.1% gelatin under nondenaturing conditions. SDS was removed by washing the gel twice for 15 min each in 50 mm Tris/HCl and 5 mm CaCl_2_ with 2.5% Triton X‐100, followed by a single wash for another 15 min in the same buffer but with 0.1% Triton X‐100. Finally, gels were incubated at 37 °C for 16 h in buffer containing 0.1% Triton X‐100 and bands were visualized with Coomassie staining. For inhibition assays, 20 mm EDTA was added during the final incubation step.

### 2.14 Breast cancer clinical data validation

The results were validated on an independent set from The Cancer Genome Atlas (TCGA) databases (Cancer Genome Atlas Network, 2012). The breast cancer dataset was downloaded from TCGA and from cBio Cancer Genomics at the Memorial Sloan Kettering Cancer Center. Data were analyzed from 1106 breast cancer cases and processed as described earlier (Sidiropoulos *et al*., 2016).

## 3 Results

### 3.1 Experimental design

**Figure 1:**
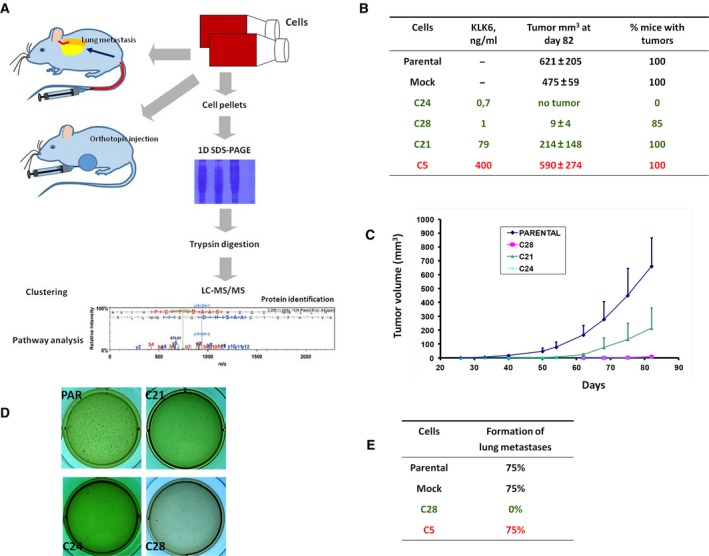
Restoration of KLK6 expression reduces the *in vitro* and *in vivo* tumorigenicity of MDA‐MB‐231 cells dose‐dependently. (A) Schematic representation of the experimental design followed in the present study. (B, C) *In vivo* growth rate and tumor volume of MDA‐MB‐231 cells injected into mouse mammary fat pad. Bars indicate SD. C5 clone has been described previously (Pampalakis *et al*., 2009). (D) Anchorage‐independent growth assay indicates that only PAR cells and to a lesser extent C21 cells have the ability to form colonies when grown on soft agar. (E) PAR, MOCK, and C5 cells can form tumors in mouse lungs when i.v. injected.

The experimental approach followed is depicted in Fig. 1A. MDA‐MB‐231 cells were stably transfected with the expression plasmid pcDNA3.1(+)/preproKLK6 cDNA that encodes the full‐length KLK6 protein. Stably transfected clones were selected and established in culture. KLK6 expression was measured in cell culture supernatants using a KLK6‐specific ELISA (Diamandis *et al*., 2000). Phenotypic characterization included the ability of cells to (a) form colonies on soft agar, (b) form tumors when orthotopically implanted in SCID mice, and (c) colonize the lungs. MDA‐MB‐231 cells were selected because they do not express any KLKs, and re‐expression of KLK6 did not induce the expression of any other KLKs (data not shown), which allowed us to dissect the function of KLK6 from potential overlap with functions from other KLKs.

### 3.2 Dose‐dependent effect of KLK6 on the tumorigenic phenotype of MDA‐MB‐231 cells

The dose‐dependent effect of KLK6 has been documented previously (Pampalakis *et al*., 2009). Here, we included cells that express intermediate KLK6 levels (C21) and new cells that express physiological levels (C28 and C24). C5 cells, the KLK6‐overexpressing cells, have been described previously (Pampalakis *et al*., 2009). We verified that re‐expression of KLK6 inhibits the tumorigenicity of MDA‐MB‐231 cells. C21 cells are associated with relatively aggressive phenotype supporting the dose‐dependent effect of KLK6 on breast malignancy in a more detailed manner (Fig. 1B–D). The ability of KLK6‐expressing cells to metastasize was investigated with tail vein assay. C5 cells metastasize to lungs at similar efficiencies as PAR cells, while C28 cells do not metastasize (Fig. 1E). Therefore, KLK6 affects the ability of breast cancer cells to colonize the lungs dose‐dependently.

### 3.3 Proteomic profiling

**Figure 2:**
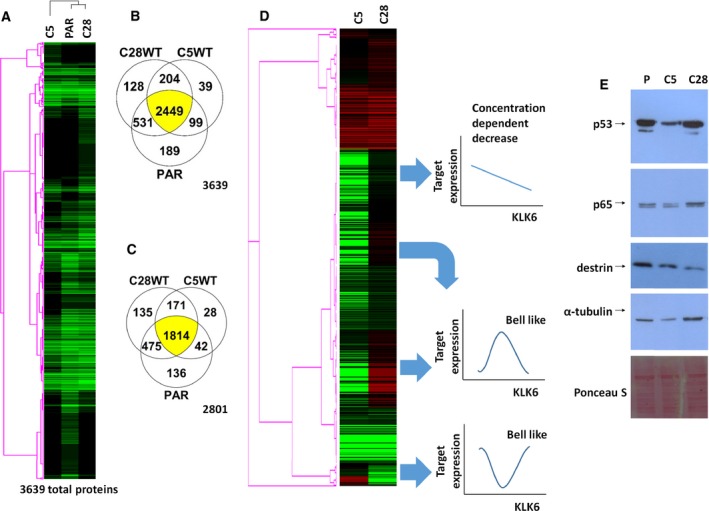
Proteomic profiling of MDA‐MB‐231 cells. (A) In total, 3639 proteins in PAR, C5, and C28 cells were identified. (B) Venn diagram shows the common proteins between the different clones. (C) Venn diagram shows the common proteins identified with ≥ 95% probability (total 2801 proteins) between the different clones. (D) Differential proteomic profiling. Black depicts no expression, green lower expression, and red higher expression compared to PAR cells. (E) Western blot analysis of selected proteins (p53, p65, destrin, and α‐tubulin) verifies the proteomic profiling.

Parental (KLK6 nonexpressing), C5 (KLK6 highly overexpressing), and C28 (KLK6 physiological‐expressing) MDA‐MD‐231 cells were selected for proteomic profiling to identify molecular pathways that determine their distinct tumor characteristics in relation to KLK6 levels. We collected 146 947 spectra from C28, 114 113 from C5, and 135 382 from PAR cells. The average spectral counts were 24.94 for C28, 16.54 for C5, and 20.56 for PAR. In total, 3639 proteins were identified, and 2449 were present in all three samples (Fig. 2A,B; Table S1). The number of proteins is distributed as follows: 3268 proteins in PAR, 3312 in C28, and 2731 in C5. With ≥ 95% probability, we found 2801 proteins: 2407 in PAR, 2553 in C28, and 1983 in C5. From this list, 1814 proteins were present in all cells (Fig. 2C), while 2341 were found to display at least twofold difference (up or down) in either C5 or C28 cells compared to PAR. The identified proteins show approximately the same distribution in terms of function and subcellular localization in all cells (PAR, C5, and C28; Fig. S1). The relative expression levels between proteins found in C5 or C28 compared to PAR are shown in Fig. 2D. Protein expression changes can be grouped in three categories: (a) proteins that remain almost unaffected upon KLK6 re‐expression independently from the levels of KLK6 expression, (b) proteins with expression that is decreased or increased in a KLK6 concentration‐dependent manner, and (c) proteins that display bell‐shaped expression. The last category could explain why KLK6 exerts different effects on the MDA‐MB‐231 phenotype when re‐expressed at physiological levels relative to overexpression. Proteomic profiling results were validated by western blot analysis of p53, p65, α‐tubulin, and destrin (Fig. 2E).

**Figure 3:**
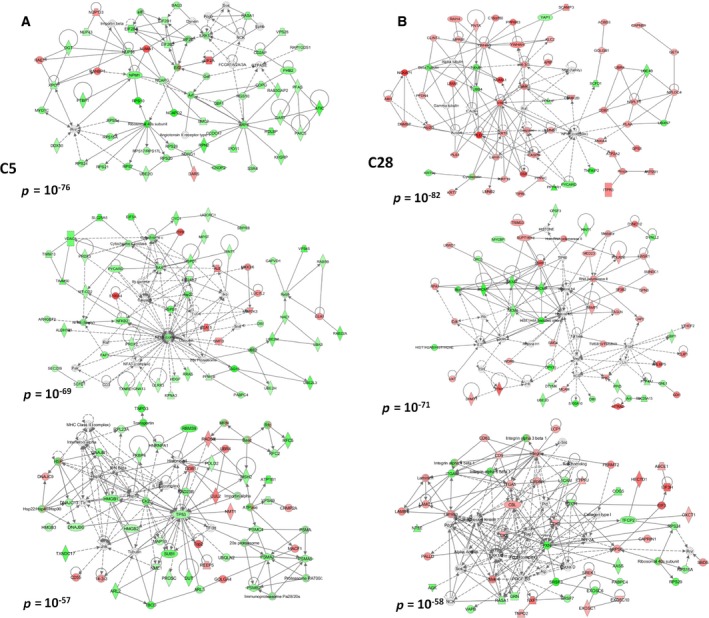
Molecular pathways. (A) Networks constructed based on the proteome of C5 cells or on the proteome of C28 cells. (B) The first three networks (lower *P* values) generated by ipa are depicted.

We then searched for pathways that are differentially activated or inactivated between C5 and C28 cells. For the generation of pathways (Fig. 3), only proteins displaying ≥ 4‐fold changes between C5 and PAR, or C28 and PAR, were analyzed with ipa.

### 3.4 Re‐expression of KLK6 at physiological levels reduces the expression of S100 proteins

**Figure 4:**
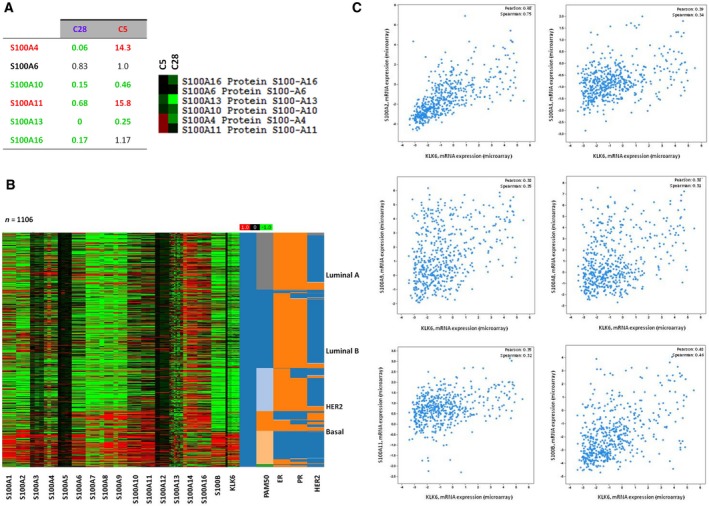
KLK6 affects the levels of S100A proteins. (A) Proteomic data indicate that in C28 cells, there is downregulation of S100A proteins, while in C5 cells, there is upregulation of S100A4 and S100A11. (B, C) *In silico* correlation of S100 expression with KLK6 expression in breast cancer clinical specimens.

Multiple members of the S100 family are upregulated in cancer. Overexpression of S100A1, A4, A6, A7, A8, A9, A11, and A14 in breast cancer correlates with aggressive phenotype (Bresnick *et al*., 2015). S100A4 was studied extensively and correlated with metastatic disease and poor survival (Bresnick *et al*., 2015). S100A proteins were downregulated in C28 cells, while in C5, S100A4 and S100A11 were upregulated (Fig. 4A). This bell‐shaped expression pattern of S100 proteins relative to KLK6 expression could explain why C5 cells have the ability to metastasize to lungs in contrast to C28. The correlation of KLK6 expression with S100 proteins was further investigated in clinical datasets from breast cancer patients. Physiological KLK6 levels inversely correlated with S100A11 and S100A14 (Fig. 4B,C). In basal‐like cancer, there was a positive correlation of KLK6 and S100A11 and S100A2 (Fig. 4B,C), in accordance with our findings.

### 3.5 Physiological levels of KLK6 induce the expression of apoptosis‐related proteins

**Figure 5:**
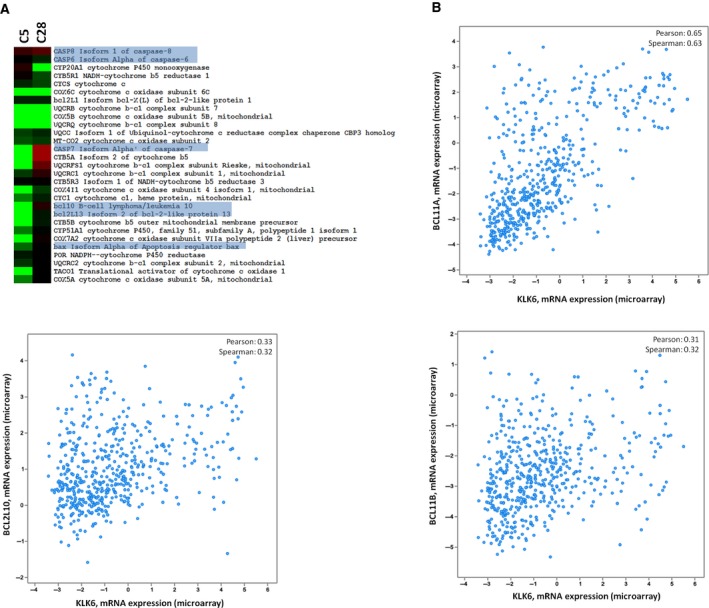
KLK6 affects the apoptosis‐related genes. (A) Expression of apoptosis‐related proteins in C28 cells and C5 cells (relative to PAR). (B) *In silico* correlation of apoptosis‐related genes with KLK6 expression in breast cancer clinical specimens.

Apoptosis‐related proteins were upregulated in C28 but downregulated in C5 cells (Fig. 5). The increased expression of apoptosis‐mediating proteins such as CASP8 and CASP7 in C28 cells may account for their reduced tumorigenicity. The association of KLK6 overexpression with apoptosis is also demonstrated by the fact that many members of the annexin family are reduced in C5 compared to C28 or PAR cells (Table S1). Consistently, it was previously reported that high levels of KLK6 promote resistance to apoptosis in A549 lung cancer cells (Michel *et al*., 2014). Analysis of breast cancer clinical datasets showed correlation of KLK6 expression with the expression of BCL members (Fig. 5).

### 3.6 Re‐expression of KLK6 reduces the expression of RAB proteins

**Figure 6:**
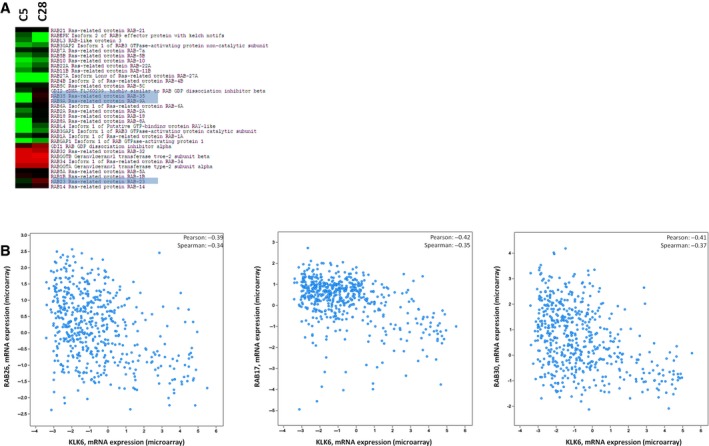
KLK6 affects the expression of RAB proteins. (A) Expression of RABs in C28 cells and C5 cells (relative to PAR). (B) *In silico* correlation of RAB gene expression with KLK6 expression in breast cancer clinical specimens.

RABs are intracellular transport proteins that regulate vesicle trafficking and intracellular communication. Vesicle trafficking is important to epithelial carcinogenesis since aberrant trafficking has been linked to loss of cell polarity, increased proliferation, cellular motility, and invasion. Altered expression of multiple members of RABs has been observed in various cancers (Goldenring, 2013). The expression of RABs in MDA‐MB‐231 showed high variability: Some members exhibited slight reduction in C28 but more pronounced in C5 cells, while others exhibited increased levels in both C5 and C28 cells (Fig. 6). Changes in RAB expression indicate reorganization of vesicle trafficking in C5 and C28 cells, and RABs that are severely downregulated in C5 but remain almost unchanged in C28 (shaded box in Fig. 6) could associate with increased metastatic ability and tumorigenicity. As shown in Fig. 6, RAB23 is upregulated in C28 but downregulated in C5 cells. Consistently, it has been shown that overexpression of RAB23 in MDA‐MB‐231 causes cell cycle arrest, inhibits growth and proliferation, and induces apoptosis (Liu *et al*., 2015). RAB35 that displays a similar pattern to RAB23 promotes cell–cell adhesion (Allaire *et al*., 2013). Integrin β1 reported to be suppressed by RAB35 (Allaire *et al*., 2013) was found slightly upregulated in C5 (spectral counts in C28: 51; C5: 109; and PAR: 73). *In silico* analysis of breast cancer clinical datasets showed that KLK6 expression inversely correlates with RAB26, RAB17, and RAB30 (Fig. 6). However, we could not associate RAB35 or RAB23 expression with KLK6 expression in clinical samples. This fact could indicate that this correlation is restricted to MDA‐MB‐231. Nonetheless, it should be noted that bioinformatic analysis was based on available transcriptomic data, which do not always overlap with proteomics.

Macropinocytosis is considered to drive the uptake of prion and SOD1 aggregates associated with neurodegenerative disorders (Creutzfeldt–Jakob disease and amyotrophic lateral sclerosis, respectively) by neuronal cells. The cellular proteins that regulate macropinocytosis were recently described and shown to be cofilin‐1, ROCK1, RAB5C, and RAB10 (Zhong *et al*., 2018). All these proteins were downregulated in KLK6‐expressing cells [cofilin‐1 is reduced in both C28 and C5 (spectral counts: C28: 282; C5: 127; PAR: 404), ROCK1 (C28: 0; C5: 0; PAR: 2), RAB5C (C28: 23; C5: 25; PAR: 44), and RAB10 (C28: 8; C5: 2; PAR: 59)]. Therefore, the KLK6‐expressing cells may have reduced ability for macropinocytosis. This could explain our recent finding that *Klk6* ^*−/−*^ primary neuronal cells display increased ability to uptake α‐synuclein aggregates with prion‐like propagation properties compared to wild‐type neuronal cells (Pampalakis *et al*., 2017).

### 3.7 KLK6 is coordinately expressed with multiple keratins

Keratins (KRTs) participate in EMT and could be involved in cancer invasion and metastasis (Karantza, 2011). Figure S2A shows upregulation of KRT8 and KRT18 in both C5 and C28 cells in accordance with the mesenchymal‐to‐epithelial transition induced upon restoration of KLK6 expression in MDA‐MB‐231 (Pampalakis *et al*., 2009). We suggested that KLK6 upregulates KRT8 expression through the TGF‐β pathway (Pampalakis *et al*., 2008). KRT81 was upregulated in KLK6‐overexpressing C5 cells but downregulated in C28 cells expressing low KLK6. The physiological significance of this finding is not known but may indicate that KRT81 promotes tumorigenesis. Analysis of breast cancer clinical datasets showed correlation (direct or inverse) of KRT expression with KLK6 including an inverse correlation of KLK6 and KRT18 (Fig. S2B,C).

### 3.8 Re‐expression of KLK6 results in reduced levels of ribosomal proteins

The expression of ribosomal and mitochondrial ribosomal proteins (RPs) is reduced upon re‐expression of KLK6 in a dose‐dependent manner with higher KLK6 expression causing higher suppression of RPs (Fig. S3). RPs bind MDM2 and block p53 degradation (Zhang and Lu, 2009). Thus, lower levels of RPs lead to higher p53. C5 cells express low levels of RPs and low levels of p53. This discrepancy could be explained by the fact that MDA‐MB‐231 cells express mutant p53 that is not under control of MDM2 (Vijayakumaran *et al*., 2015). Analysis of RP expression in breast cancer clinical datasets did not show correlation with KLK6 expression, indicating that the described association could be restricted to MDA‐MB‐231 cells.

### 3.9 KLK6 re‐expression induces changes in secreted metalloprotease activities

Gel zymography and secretomics (Table S2) were used to analyze the expression of proteolytic enzymes in cell supernatants. As shown in Fig. S4, C28 cells expressed significantly lower amount of MMP2 and MMP9 compared to PAR and C5 cells. Therefore, re‐expression of KLK6 at physiological levels reduces the expression of gelatinases that strongly correlate with tumor metastasis and progression (Deryugina and Quigley, 2006). The higher band of MMP2 detected by western blot in C28 could be a MMP2/TIMP2 complex that is partially resistant to proteolysis. Recently, we showed that KLK6 cleaves and activates proMMP2 (Pampalakis *et al*., 2017), while another study showed that KLK6 activates proMMP9 (Bando *et al*., 2018). Therefore, in C5 cells expression and activation of proMMP2 and proMMP9 could occur and contribute to increased tumorigenicity.

### 3.10 Survival analysis

**Figure 7:**
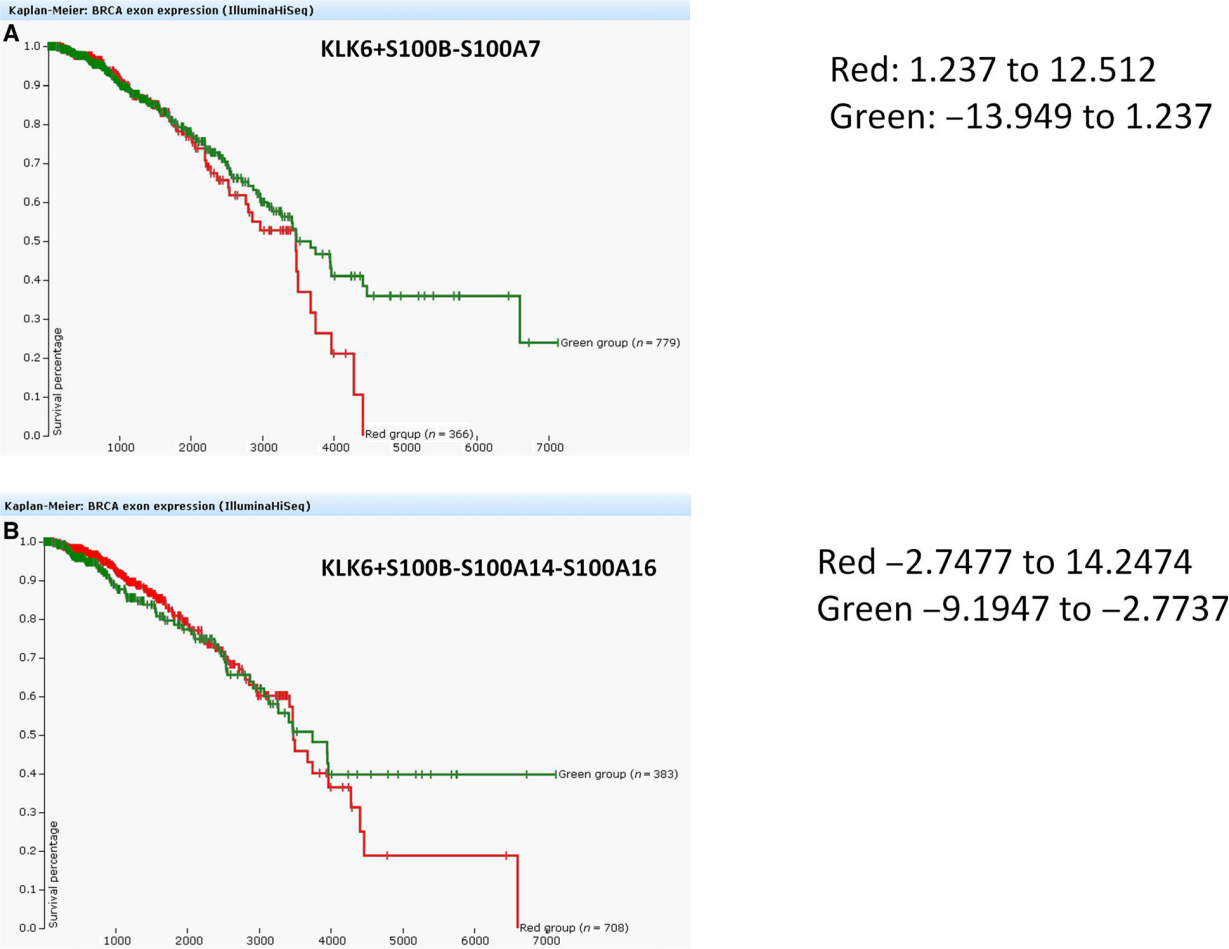
Overall survival (OS) scores. (A) KLK6 + S100B‐S100A7 scoring expression in breast cancer patients. Green indicates expression score −13.949 to 1.237; red indicates expression score 1.237–12.512. (B) KLK6 + S100B‐S100A14‐S100A16 scoring expression in breast cancer patients. Green indicates expression score −9.1947 to −2.7737; red indicates expression score −2.7477 to 14.2474.

Two composite scores, KLK6 + S100B‐S100A7 and KLK6 + S100B‐S100A14‐S100A16, were generated to predict long‐term survival of breast cancer patients (Fig. 7A,B). The generation of these scores was based on our findings and used a trial‐and‐error approach. The composite scores were equally weighted. For the KLK6 + S100B‐S100A7, breast cancer patients with the lowest scores have longer long‐term survival as compared to patients with high scores. Therefore, this score could find diagnostic applications.

## 4 Discussion

KLK6 displays aberrant expression in various types of cancer, including breast cancer (Anisowicz *et al*., 1996; Haritos *et al*., 2018; Wang *et al*., 2008), head and neck squamous cell cancer (Schrader *et al*., 2015), renal cancer (Tailor *et al*., 2018), and colon cancer (Kim *et al*., 2011; Petrak *et al*., 2012). Very recently, we showed that KLK6 accelerates nonmelanoma skin cancer formation *in vivo* by increasing tumor‐associated inflammation (Khoury *et al*., 2018). KLK6 expression in breast cancer either above or below the physiological levels has been associated with reduced overall survival of breast cancer patients (Sidiropoulos *et al*., 2016). In breast cancer, reduced *KLK6* mRNA levels have been found relative to noncancerous tissue, but in TNBC and HER2‐positive cancer, there is an overexpression of *KLK6* mRNA and *KLK6* levels constitute an unfavorable marker for breast cancer (Haritos *et al*., 2018). Also, the expression of KLK6 is significantly higher in the serum of breast cancer patients compared to healthy individuals (Mangé *et al*., 2016).

Using proteomics, we show that physiological levels of KLK6 in C28 clone are associated with increased expression of apoptosis‐mediating proteins such as CASP8 and CASP7, while high levels of KLK6 are associated with high expression of S100 proteins. These differentially expressed proteins could explain why C28 cells have reduced tumorigenicity while C5 cells display a more aggressive phenotype and increased ability to form lung metastases. It is known that S100 proteins are upregulated in basal‐like breast carcinomas and increased expression of S100A11 and S100A14 was correlated with poor outcome in breast cancer (McKierman *et al*., 2011). S100A4 mediates breast cancer metastasis, and its expression is controlled via the β‐catenin pathway (Bresnick *et al*., 2015); therefore, high levels of KLK6 could activate the β‐catenin pathway as reported in keratinocytes (Klucky *et al*., 2007), to drive the expression of S100A4. S100A4, in turn, enhances the expression of MMP9 and TIMP1 at the transcriptional level thus increasing tumorigenicity (Saleem *et al*., 2006). S100A4 was also shown to increase MMP2 levels in MDA‐MB‐231 and MDA‐MB‐468 breast cancer cells (Xu *et al*., 2015). Consistently, we found increased expression of MMP9 in the secretome of C5 cells but reduced expression in C28 cells in which KLK6 is expressed at low levels.

Although C5 cells display increased tumorigenicity, they have reduced levels of mutant p53 (R280K). In MDA‐MB‐231, mutant p53 (R280K) regulates the expression of genes encoding enzymes of the mevalonate pathway. shRNA‐mediated knockdown of mutant p53 downregulates the mevalonate pathway and alters cellular morphology to more acinar‐like morphology reducing their tumorigenicity (Freed‐Pastor *et al*., 2012). Therefore, overexpression of KLK6 (clone C5) could reduce the expression of mutant p53 to increase tumorigenicity. In addition, mutant p53 downregulates Dicer expression (Muller *et al*., 2014). Since C5 cells express low levels of p53, this explains our recent observation that C5 cells express high levels of Dicer mRNA (Sidiropoulos *et al*., 2016).

Caspases have tumor suppressor functions, and in breast cancer cells, downregulation of CASP8 has been reported (Olsson and Zhivotovsky, 2011). Therefore, upregulation of CASP8 and CASP7 in C28 cells could account for their reduced tumorigenicity by inducing apoptosis. Loss of cell polarity is characteristic of epithelial carcinogenesis. RAB proteins are membrane trafficking regulators that are responsible for cellular polarity (Goldenring, 2013). Both C28 and C5 expressed reduced levels of the RABs, and this was more pronounced in C5 although we did not detect changes in RAB5A that has been linked to breast cancer metastasis (Goldenring, 2013).

KLK6 has been implicated in EMT in breast cancer (Pampalakis *et al*., 2009), in gastric cancer by reducing the expression of E‐cadherin at the transcriptional level (Kim *et al*., 2012), and in colon cancer (Kim *et al*., 2011). Finally, KLK6 may regulate the TGF‐β pathway (Pampalakis *et al*., 2008, 2017; Qin *et al*., 2012). How KLK6 expression is regulated in breast cancer is not fully elucidated. KLK6 silencing in breast cancer cells occurs through promoter methylation (Pampalakis *et al*., 2009). On the other hand, RhoA upregulates the expression of KLK6 at the transcriptional level (Teo *et al*., 2016). Since RhoA is expressed in MDA‐MB‐231 (Table S1), it could be that the endogenous KLK6 promoter methylation prevents its expression. KLK6 is regulated by mutant K‐RAS in colon cancer cells to enhance their migration and invasion (Henkhaus *et al*., 2008). Recently, KLK6 was shown to post‐transcriptionally process 6‐phosphofructo‐1‐kinase (PFK‐M) to produce shorter highly active fragments, while it increased the growth rate of *Saccharomyces cerevisiae* engineered to express human PFK‐M. Therefore, KLK6 could trigger glycolytic flux in cancer cells (Andreic and Legiša, 2018). Our data did not find the KLK6‐proteolytic fragments of PFK‐M, but this could be due to their very low expression compared to other peptide fragments.

## 5 Conclusion

In conclusion, we showed that KLK6 displays a concentration‐dependent effect on breast primary tumor growth and metastasis. Specifically, when KLK6 is expressed at levels comparable to the levels secreted by normal mammary cells, it inhibits primary tumor formation and lung metastasis *in vivo*. On the other hand, overexpression of KLK6 (> 50‐fold higher than normal), characteristic of a subset of breast cancers, promotes primary tumorigenesis and lung metastasis *in vivo*. For the first time, we correlated tumorigenicity of KLK6 with the expression of S100A and apoptosis‐mediated proteins. Based on these findings, we provided a combined score that could assist in the prognosis of breast cancer patients. Finally, as mentioned previously the regulation of RABs by KLK6 could be responsible for the enhanced uptake of α‐synuclein aggregates from the *Klk6* ^*−/−*^ cells, a finding that merits further investigation in the future. Thus, whether the identified KLK6‐regulated proteins and corresponding pathways operate in other systems besides breast cancer remains to be investigated.

## Conflict of interest

The authors declare no conflict of interest.

## Author contributions

GP performed experiments and wrote the manuscript; EZ performed experiments; KGS and GMY analyzed the clinical data; EPD acquired MS data; VZ performed experiments with animals; and GS conceived, designed, and supervised the project, analyzed the data, acquired funding, and wrote the manuscript.

## Supporting information


**Fig. S1.** Classification of the identified proteins according to Gene Ontology (GO). Pie chart shows the classification of proteins according to the biological processes (A) and cellular component (B). Bar chart shows the distribution of proteins according to the biological processes (C) and cellular component (D) for each clone (PAR, C5, and C28).

**Fig. S2.** Association of KLK6 and keratins. treeview (A) and list of KRTs (B) and their expression in C28 and C5 cells. Red color indicates higher expression than PAR and green color lower expression. C, *In silico* correlation of KRT expression with KLK6 expression in breast cancer clinical specimens.

**Fig. S3.** KLK6 affects the expression of ribosomal proteins. treeview of ribosomal and mitochondrial ribosomal proteins expressed in C28 and C5 cells. Red color indicates higher expression than PAR and green color lower.

**Fig. S4.** KLK6 expression affects the levels of metalloprotease expression. A, Gelatin zymography of SFCMs obtained from PAR (P), C5, and C28 cells. B, Western blot analysis of MMP9 and MMP2 expression in SFCMs from PAR (P), C5, and C28 cells. C, Expression of MMPs and their inhibitors in SFCMs identified by HPLC‐MS/MS analysis. The numbers on the left indicate the fold increase in C5 SFCM relative to PAR SFCM (P). Secretomics were conducted on SFCMs from C5 and PAR cells. For this, after carefully washing the cells (3 times with PBS and 3 times with SFCM), new SFCMs were added in cells for 48 h. The SFCMs were collected and centrifuged to remove cell debris and stored at −80 °C until the analysis. The detailed secretomic profiling is given in Table S2.

**Table S1.** Results from proteomic profiling.

**Table S2.** Results from secretomic profiling.
