# Supplementary figures and images for: Biochemical pathways mediated by KLK6 protease in breast cancer
Source: Mol Oncol. 2019 Sep 30;13(11):2329–43. doi: 10.1002/1878-0261.12493 (PMC6822253; doi:10.1002/1878-0261.12493)

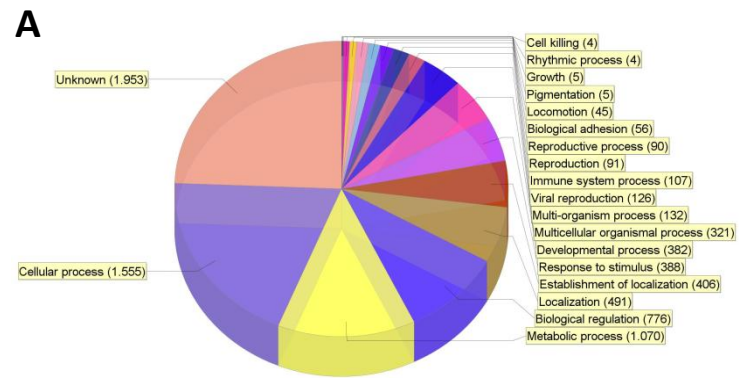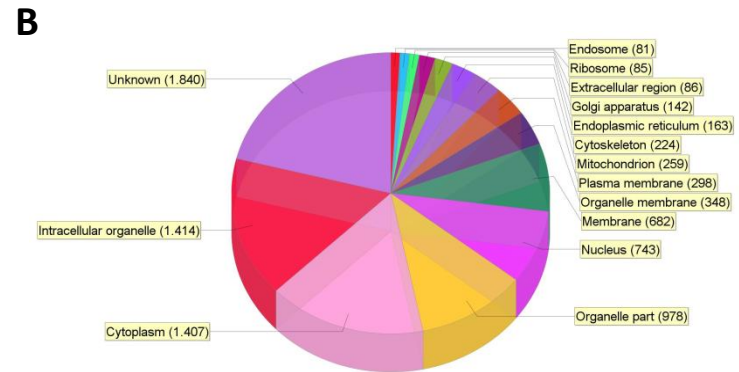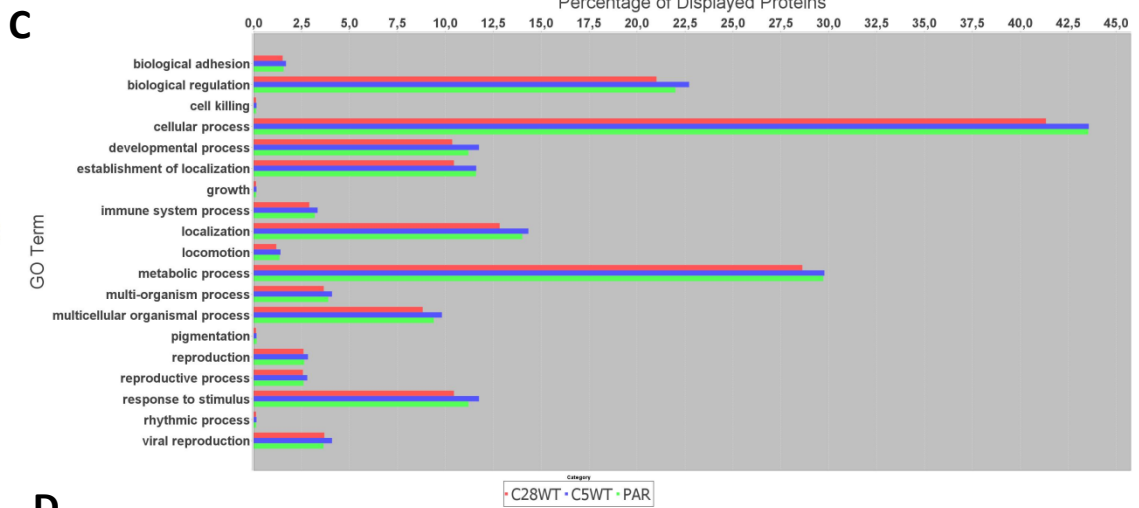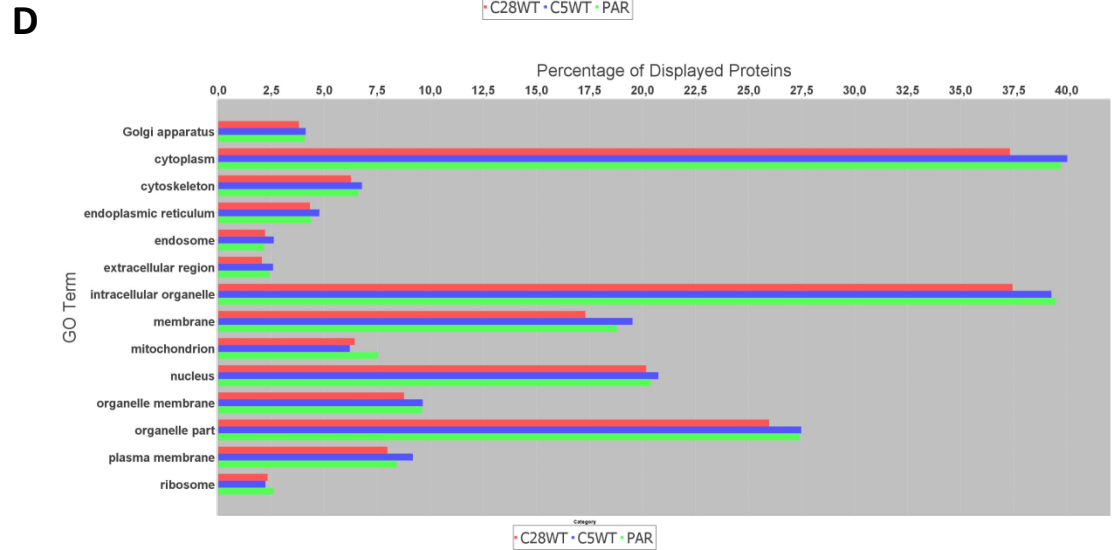

**Figure S1**

Supplement: Supplementary file 1 — Fig. S1. Classification of the identified proteins according to Gene Ontology (GO). Pie chart shows the classification of proteins according to the biological processes (A) and cellular component (B). Bar chart shows the distribution of proteins according to the biological processes (C) and cellular component (D) for each clone (PAR, C5, and C28). [file MOL2-13-2329-s001.pdf]

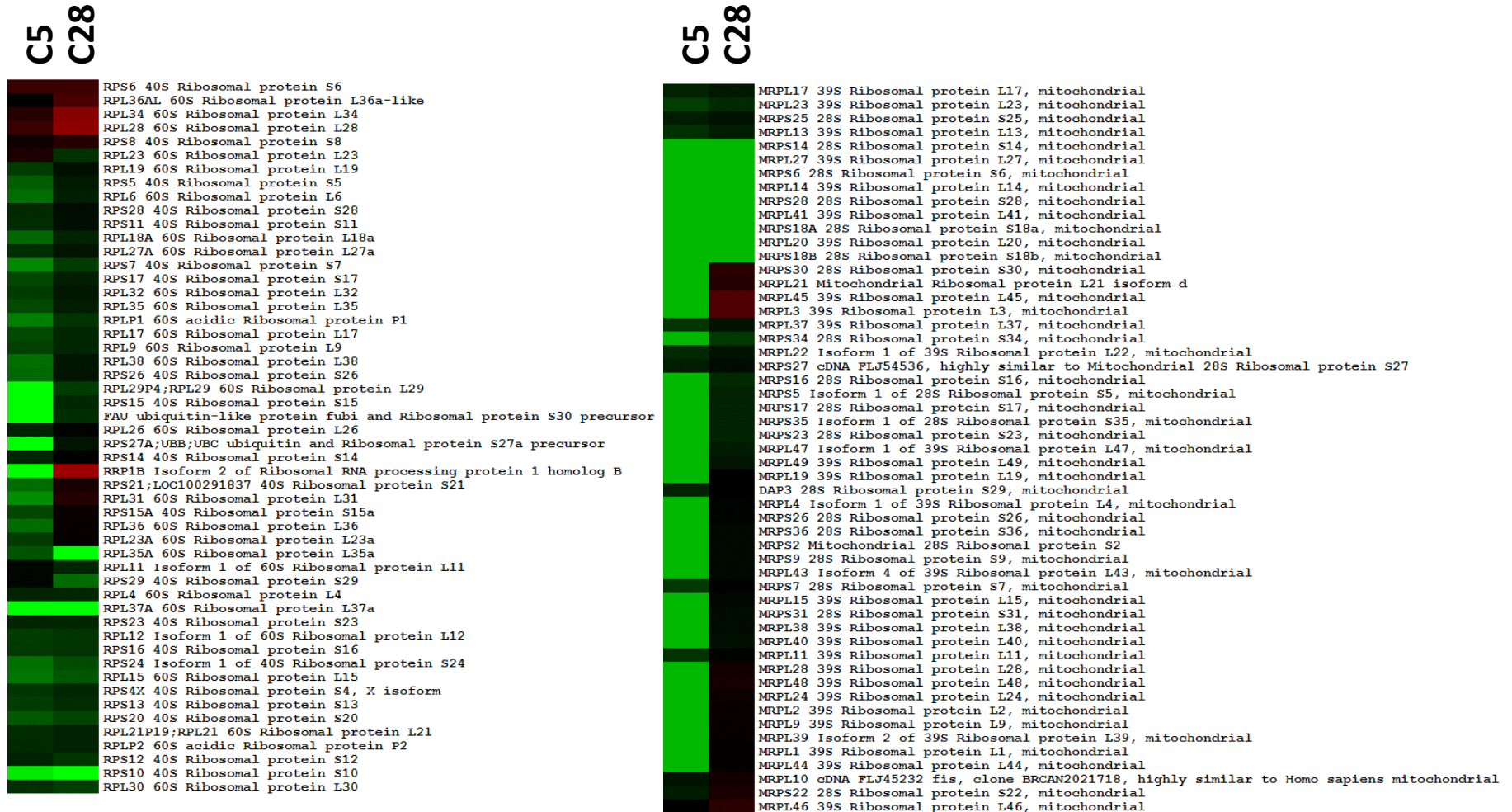

**Figure S3**

Supplement: Supplementary file 3 — Fig. S3. KLK6 affects the expression of ribosomal proteins. treeview of ribosomal and mitochondrial ribosomal proteins expressed in C28 and C5 cells. Red color indicates higher expression than PAR and green color lower. [file MOL2-13-2329-s003.pdf]

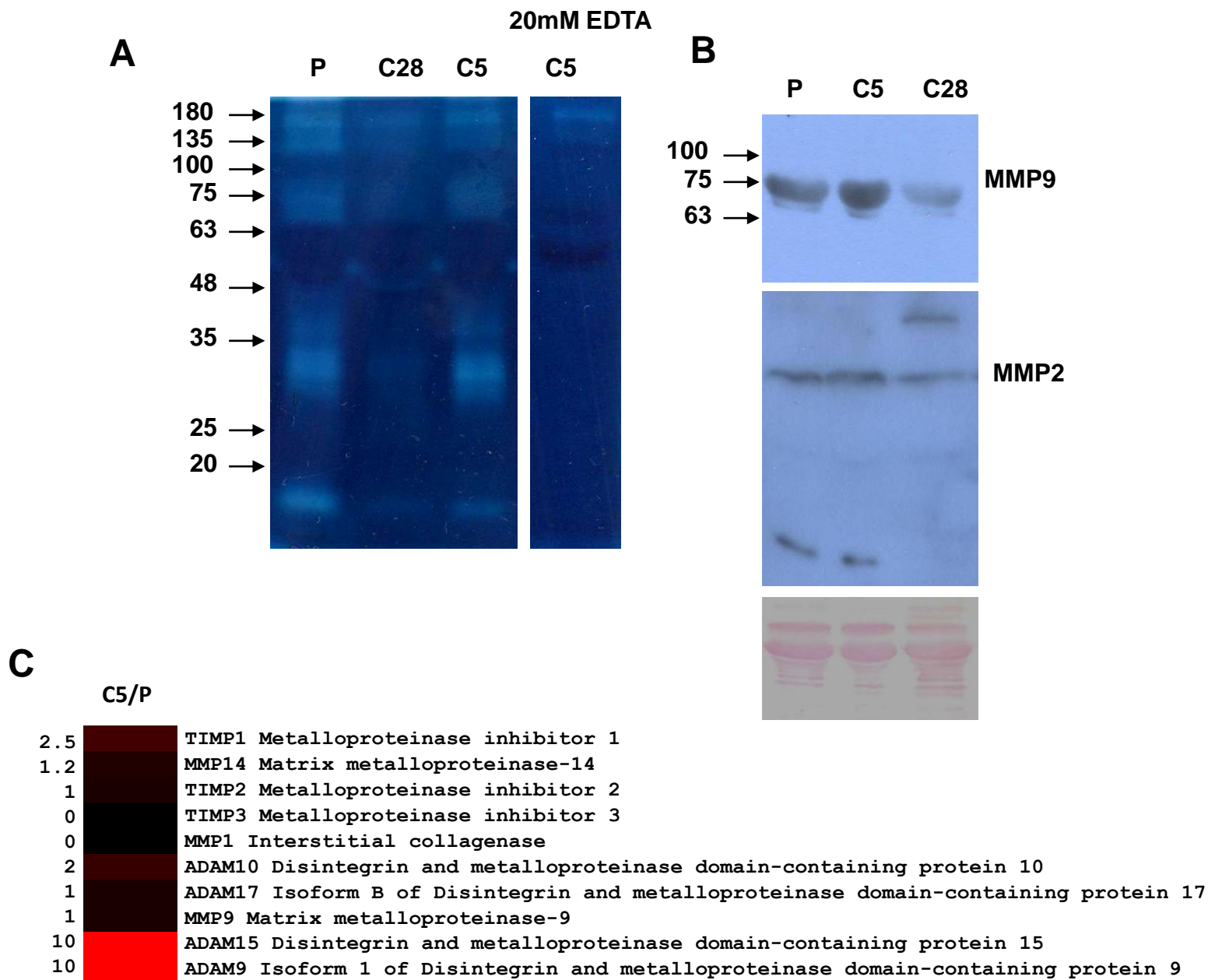

**Figure S4**

Supplement: Supplementary file 4 — Fig. S4. KLK6 expression affects the levels of metalloprotease expression. A, Gelatin zymography of SFCMs obtained from PAR (P), C5, and C28 cells. B, Western blot analysis of MMP9 and MMP2 expression in SFCMs from PAR (P), C5, and C28 cells. C, Expression of MMPs and their inhibitors in SFCMs identified by HPLC‐MS/MS analysis. The numbers on the left indicate the fold increase in C5 SFCM relative to PAR SFCM (P). Secretomics were conducted on SFCMs from C5 and PAR cells. For this, after carefully washing the cells (3 times with PBS and 3 times with SFCM), new SFCMs were added in cells for 48 h. The SFCMs were collected and centrifuged to remove cell debris and stored at −80 °C until the analysis. The detailed secretomic profiling is given in Table S2. [file MOL2-13-2329-s004.pdf]
