# Supplementary material for: Biochemical pathways mediated by KLK6 protease in breast cancer
Source: Mol Oncol. 2019 Sep 30;13(11):2329–43. doi: 10.1002/1878-0261.12493 (PMC6822253; doi:10.1002/1878-0261.12493)
Supplement: Supplementary file 2 — Fig. S2. Association of KLK6 and keratins. treeview (A) and list of KRTs (B) and their expression in C28 and C5 cells. Red color indicates higher expression than PAR and green color lower expression. C, In silico correlation of KRT expression with KLK6 expression in breast cancer clinical specimens. [file MOL2-13-2329-s002.pdf]

A

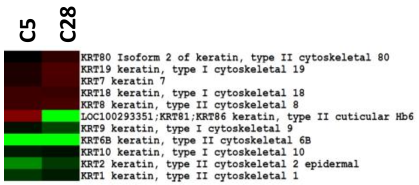

B

Keratin	Pearson Correlation	Spearman Correlation
KRT17	0.77	0.83
KRT6B	0.69	0.81
KRT6A	0.74	0.81
KRT6C	0.74	0.81
KRT5	0.70	0.79
KRT14	0.67	0.78
KRT16	0.72	0.73
KRT15	0.44	0.48
KRT13	0.40	0.46
KRT81	0.49	0.45
KRT75	0.41	0.39
KRT23	0.43	0.38
KRT37	-0.40	-0.36
KRT18	-0.46	-0.34
KRT7	0.35	0.33
KRTAP6-3	0.33	0.32
KRT83	0.43	0.30

C

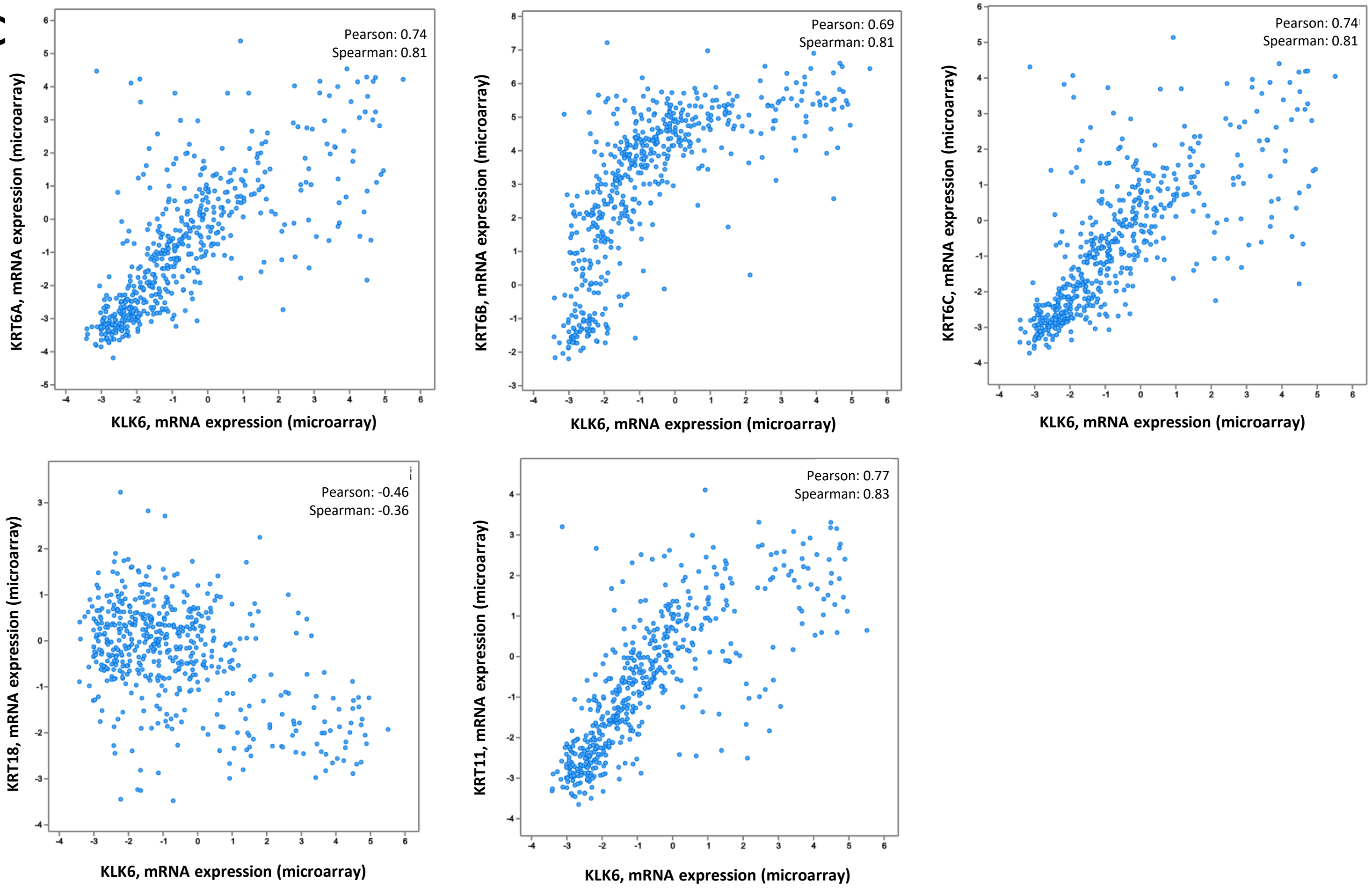

Figure S2
